# Toward a knowledge economy: Factors affecting the sustainable consumption behavior in the Chinese online education industry

**DOI:** 10.3389/fpsyg.2022.1007230

**Published:** 2022-11-21

**Authors:** Ruihui Pu, Songyu Jiang, Rebecca Kechen Dong, Thitinan Chankoson, Adul Supanut, Suppanunta Romprasert, Danai Tanamee

**Affiliations:** ^1^Faculty of Economics, Srinakharinwirot University, Bangkok, Thailand; ^2^Pass College of Chongqing Technology and Business University, Chongqing, China; ^3^UTS Business School, University of Technology Sydney, Sydney, NSW, Australia; ^4^Faculty of Business Administration for Society, Srinakharinwirot University, Bangkok, Thailand

**Keywords:** online education for sustainability, digital economy, sustainable consumption behavior, sharing economy, online education industry, sustainable development goals

## Abstract

**Introduction:**

Buoyed by recent calls to research and advance the knowledge economy and sustainable development. This study explains how the role of the knowledge economy in influencing the COVID-19 pandemic has emerged with numerous opportunities for the global E-learning or online education industry. And, knowledge sharing behavior has been hugely driven by various sharing platforms concerning a new paradigm for diversifying education and learning. However, our study is to further extend the understanding and examine the related empirically correlations to deepen online education for sustainable development (OESD). Both advancing theoretical underpinnings and enhancing the online education industry are highly integrated and introduced toward a sustainable pathway. This study brings the perspectives from consumer value, social identity social exchange, and value-attitude-behavior to explain sustainable consumption behavior in the Chinese online education industry (SCBOEI). Thus, the relationship among factors in this study is statistically examined and the SCBOEI model as the new theoretical insight is introduced in a way of sustainable consumption behaviors in the Chinese online education industry. Finally, this study addresses managerial implications to practitioners, the government, universities, and markets.

**Materials and methods:**

Employing a quantitative approach, about 559 valid questionnaire surveys are collected from Chinese higher education institutions. This study includes participants from four controlling variables (age, education level, family income, and gender) and six latent variables. The bootstrapping method was applied to validate mediating factors and their interacting relationships.

**Results:**

The finding reveals that a set of classic psychological theories related fits the SCBOEI in higher education from the consumer value, contextual factors, social identity, sustainable consumption attitudes, and consumer engagement to explain SCBOEI. The mediating role of identity, sustainable consumption attitudes, and consumer engagement is highly concerned. The value and contextual factors directly make impact on SCBOEI through identity, sustainable consumption attitudes, and consumer engagement.

**Implications:**

The study significantly contributes to enriching the theoretical bases for advancing the literature on sustainable consumption behavior and online education. Our research provides managerial insights into government policy about the online education industry and marketers to set more advertisements to wake awareness of SCBOEI. Furthermore, higher education institutions should encourage their employees and students to participate in SCBOEI actively. All the stakeholders are essential to lead the consumer to SCBOEI by shaping their internal psychology while paying more attention to social equality (education, gender), responsible consumption, and decent economic development. Overall, addressing these issues will help to provide scholars with novel theoretical insights and practitioners with managerial advice.

## Introduction

The emerging knowledge economy facilitates innovation and information sharing with insightful implications for the environmental, social, and governance (ESG) (Pu et al., 2022a). Most recently, there has been a growing emphasis on how the knowledge economy could bring comprehensive contributions to the online education sector. Digitizing has made a massive change in the education industry. Moreover, online education has become a novel educational paradigm due to digital technology and economy (Jiang et al., 2022). The COVID-19 pandemic has accelerated the development of the online education industry (Wargadinata et al., 2020). Nevertheless, despite an increasing interest in online education development during COVID-19, recent calls in this literature emphasize the need for a better understanding of sustainable development goals (SDGs). Organizations in diverse industries pursue sustainable consumption behavior to pave the way for SDGs (ElMassah and Mohieldin, 2020). Traditionally, sustainable consumption behavior studies focus on potential and environment-related areas such as natural energy and resource, agriculture, transportation, and tourism (Zhou et al., 2022). In recent years, with the promotion of the 5G network, the digitized economy and sharing economy have been fully developed. With this in mind, sustainable consumption behaviors are in the shared accommodation and peer-to-peer (P2P) (Pu and Pathranarakul, 2019). The online education industry, as a novel research paradigm, has become influential in achieving SDGs. Jiang and Pu (2021) pointed out that SCBOEI differs from traditional sustainable research. SCBOEI aims to resolve key issues from multiple aspects, including education equality, gender equality, knowledge sharing, employment, international educational cooperation, technological development, and responsible consumption, which are conducive to achieving sustainable development goals. On the contrary, the contribution of SCBOEI to the SDGs in the environmental dimension is inadequate. There is no doubt that SCBOEI promotes the benign development of the network education industry. At the same time, it also promotes regional economic and socially sustainable development.

As a highly liberal and open city in mainland China, Chongqing plays a vital role in constructing “*One Belt and One Road, Yangtze River Economic Zone, and Chengdu-Yu Economic Circle*” (Gong et al., 2021). Chongqing has the largest resident population in China, with more than one million college students in 2022, forming a considerable network education market potential. The local government also actively promotes the digitization of higher education and the development of network education platforms and courses and standardizes the network education market (Jiang and Pu, 2021). Therefore, the empirical investigation from the network education in Chongqing will lead to insightful and representative findings.

Although SCBOEI has essential research value, there are limited achievements in the field of online education. The main dimensions are value (functional value, social value, psychological value, and emotional value), identity, and contextual factors (government action, market conditions, education, social media, COVID-19 pandemic, and learning environment). In contrast, the environmental level (environmental attitudes and values) has a limited interpretive effect on SCBOEI (Jiang and Pu, 2022). Therefore, online education has a superior market value. Few studies discuss the development of online education and the factors affecting SCBOEI. Moreover, there is little research to discuss the impact of identity, sustainable consumption attitudes, and consumer engagement on SCBOEI and even to validate their mediation role between value and SCBOEI or contextual factors and SCBOEI. We, therefore, ask the following research question: what are the factors affecting the SCBOEI and how they are interacting? Our research objectives are 3-folded accordingly.

1. Exploring the salient underlying factors affecting the SCBOEI.
2. Analyzing the relationships between the salient underlying factors and SCBOEI.
3. Offering practical implications to diverse stakeholders regarding SCOBEI implementation.

As such, this is a rather glaring deficiency in synergizing theories in the knowledge economy literature and online education literature. Based on consumer value theory, social identity theory (Hornsey, 2008), social exchange theory (Thomas and Gupta, 2021), value-attitude-behavior theory (Wang et al., 2022), and attitude-behavior-context (Xing et al., 2022), we frame the literature review and hypothesis development. These observations offer the research motivations for our study in examining six variables that are proposed to validate the factors affecting SCBOEI. Accordingly, we build up an SCBOEI model based on statistical and analytical analyses.

## Theory and hypotheses

### Theoretical approach

Focusing on the influence of psychological factors and extrinsic factors on SCBOEI, this study explores the SCBOEI grounding from classical theories. The *consumer value theory* puts forward value positively and significantly impacts sustainable consumption behavior (Lin and Huang, 2012). Value contains many dimensions. Functional, social, and emotional values are the critical premises for predicting SCBOEI. Psychological, conditional, information, and environmental values impact SCB (Lee et al., 2015).

The *social identity theory* explores identity as a psychological factor in shaping behavior (Hogg et al., 2016). In the development of social identity theory, value and context factors have become the prerequisite to affecting identity. Identity plays an intermediary role in sustainable consumption behavior as a psychological factor (Gatersleben et al., 2014). Social identity theory supports the positive effect of consumer identity on sustainable consumption (Dermody et al., 2018). Identity is a complex concept, and research on consumer behavior often divides it into social identity, place identity, and self-identity.

The *theory of planned behavior i*s an important support for predicting relationships between attitudes and behavior, which finds subjects with value-expressive attitudes exhibited significant relations between value importance and their attitudes or behavior, whereas subjects with utilitarian attitudes did not (Kim et al., 2021). The theory of planned behavior suggests that education for sustainability is critical in determining SCB (Zhang et al., 2020). The *value-attitude-behavior* model, which is the extension of the theory of planned behavior, evident value orientation continuum predicts a respondent’s attitude toward sustainability, and that the attitude fully mediated the relationship between value orientation and behavioral intention to vote for sustainable development (Cheung and To, 2019). *Attitude-behavior-context* suggested that individual attitudes and contextual factors influence sustainable consumption behavior (Xu et al., 2017).

The *social exchange theory* awakens a kind of reciprocity between people and their actions in context factors. People’s actions are usually based on the purposeful benefits to themselves, society, and others (Thomas and Gupta, 2021). In applying this same logic derived from the social exchange theory, the study suggests that contextual factors and engagement may affect the SCBOEI. As such, contextual factors may shape the mechanism between the above psychological factors and SCBOEI.

We attempt to extend this theoretical rationale by specifying the variety and nature of SCBOEI. These theoretical assumptions suggest the importance of examining psychological factors of value, identity, and attitude because these factors drive consumers’ behavior. Therefore, the study extracted value as a variable under consumer value theory. Based on the theoretical assumptions of planned behavior theory, sustainable consumption attitude and consumer engagement may be vital variables in the model. In line with the social identity theory, the study excavates the possibility of identity as an intermediary variable. Moreover, the individual’s consumption environment is a central component of directing their behavior.

### Hypothesis development

#### Value, identity, and sustainable consumption behavior in the Chinese online education industry

The *consumer value theory* suggests that value is the psychological state that consumers perceive from all aspects of the process of consumption, which is conducive to the realization of purchasing behavior (Key et al., 2020). Value is sometimes the criterion for consumer decision-making, including the combination of multiple connotations of price, role, information, consumption environment, and consumer perception (Key et al., 2020). In addition, Jiang et al. (2022) demonstrated the positive effect of some values on SCBOEI. Therefore, the study proposes:

*H1. Value positively influences SCBOEI.*

Value in different cultures differs to a large extent because of cultural dissimilarities, social systems, social class, gender, occupation, education, religion, and political orientation (Sharma and Jha, 2017). In the case of self-oriented values such as self-enrichment and accomplishment, people reflect on the approaches and objectives that are more desirable for themselves as individuals. A self-oriented individual may not engage in sustainable consumption behavior unless he has a positive attitude toward sustainability (Graf et al., 2012). Consumption values have a positive relationship with the identity of consuming sustainable products (Biswas, 2017). The values that consumers derive from sustainable consumption not only act as a motivation to consume the product but also fulfill individuals’ self-identity to be sustainable people (Ruby et al., 2020). Thus, we posit that:

*H2. Value positively affects an individual’s identity.*

Identity is significant in explaining SCB, and its influence is more substantial than attitudes and values (Dermody et al., 2015). Identity exploration is highly relevant in determining SCB. Previous sustainable psychology research examines the specific role of self-identity in predicting behavior (Gatersleben et al., 2014). Identity can intervene in the relationship between consumer psychology and behavior (Hogg et al., 2016). Social identity affects purchasing intention, while the perceived value of social development-friendly products somewhat mediates these relationships (Adam et al., 2021). Consumption values enhance SCB through place identity (Lee et al., 2015). Individuals who identify with their community are more likely to engage in SCB, which examines how consumption value contributes to identity and SCB (Gatersleben et al., 2014). Therefore, we argue:

*H3. Identity positively influences the SCBOEI.*

*H4. Identity mediates the relationship between the value and the SCBOEI.*

#### Value, sustainable consumption attitude, and sustainable consumption behavior in the Chinese online education industry

Value plays a crucial role in decision-making and is illustrated as a broad psychological construct that could affect consumers’ interests, attitudes, and behavior (ElHaffar et al., 2020). Value predicts attitudes toward specific objects to the extent that these objects help or hinder the expression of the values (Matharu et al., 2020). Thus, we posit that:

*H5: Value has a positive impact on sustainable consumption attitude.*

*H6: Sustainable consumption attitude mediates the relationship between value and SCBOEI.*

The impact of proposed attitudes on SCB in planned behavior theory seems to have become a consensus (Alam et al., 2020). The role of environmental attitudes in shaping SCB has been supported in many fields, and sustainable consumption attitudes are both environmental and macro concepts (Kosonen and Kim, 2016). The results of discussing the role of sustainable consumption attitudes on SCB are gradually emerging. The attitude-behavior-context model suggests that individuals’ attitudes and contextual factors influence sustainable consumption behavior (Xu et al., 2017). A sustainable consumption attitude is a significant explanation of why individuals do or do not engage in sustainability-orientated behavior (Zakaria et al., 2019). Thus, we posit that:

*H7. Sustainable consumption attitude has a positive impact on SCBOEI.*

#### Value, consumer engagement, and sustainable consumption behavior in the Chinese online education industry

As a branch of attitudes research, the impact of consumer engagement on SCB is also one of the critical contents of planning behavior theory (Piligrimienë et al., 2020). The more consumers perceive the brand and inter-enterprise value, the more they can establish engagement in the consumption process (Hollebeek and Macky, 2019). Consumer value shapes their participation behavior (cognitive processing, emotion, and activation), which leads to the intention of value co-creation (Nadeem et al., 2021). Even in the study of brand consumption, the results show that value consistency significantly impacts consumer participation (Islam et al., 2018).

*H8. Value positively affects consumer engagement.*

Consumer engagement can improve consumer loyalty, satisfaction, empowerment, contact, emotional bond, trust, and commitment (Piligrimienë et al., 2020). Positive engagement is more accessible for higher education students to produce sustainable, practical behavior (Kadic-Maglajlic et al., 2019). Sustainable engagement enables consumers to actively practice sustainable consumption, which helps to solve current global challenges (Čapienë et al., 2021). Thus, this research put forward:

*H9. Consumer engagement positively affects the SCBOEI.*

Consumer engagement is an intermediary between consumer value and creative behavior (Nadeem et al., 2021). Consumers’ concept of sustainable development and engagement in sustainable consumption affect their willingness to consume (Gomes et al., 2022). Thus, this research put forward:

*H10. Consumer engagement mediates the relationship between the value and SCBOEI.*

#### Contextual factors, sustainable consumption attitude, and sustainable consumption behavior in the Chinese online education industry

Contextual factors are essential to cause SCB because the importance of social identity significantly affects decision-making (Wang et al., 2014). Contextual factors also affect learners’ adaptability to the online learning mode (Yang and Pu, 2022). With this notion in mind, we argue that higher education for sustainability predicts SCB compared to other demographic characteristics and psychological attributes (Pu et al., 2022b). Thus, the study makes a hypothesis:

*H11. Contextual factors positively affect the SCBOEI.*

In the context of sustainable development, for higher education students, education and parental influence play an essential role in sustainable consumption attitudes (Lee, 2014). Sharing attitude is gradually established, which provides internal strength for resource sharing in sustainable development. In the era of rapid technological development in the online education industry, the factors affecting the sustainable consumption attitude of consumers in online education are also diverse (Bauer et al., 2018). Thus, the study makes the following hypothesis:

*H12. Contextual factors positively affect the sustainable consumption attitude.*

In the context of social media, attitude is in the middle as a transitional factor, which has witnessed how social media shape sustainable consumption behavior and emphasizes the positive role of social media on consumers’ attitudes, thus affecting behavior (Teng et al., 2015). The context shapes a strong attitude, positively impacting behavior change (Bechler et al., 2021). The sharing economy platform positively impacts the attitude of tourism consumers, which determines the persistence and transformation of tourism consumers’ behavior in this field (Tajeddini et al., 2021).

*H13. A sustainable consumption attitude mediates the relationship between the contextual factors and SCBOEI.*

Engagement is a form of social and interactive behavior, described as a transitional state formed by developing relevant engagement processes over time (Estrada et al., 2021). The interpretation of this concept usually depends on the object of participation (company, product, brand, advertising, virtual community, and value creation) (Piligrimienë et al., 2020). Consumer engagement is the physical and emotional relationship between consumers and enterprises, retailers, and products established through interaction and cooperation in specific consumption situations (Piligrimienë et al., 2020). Consumer engagement is a vital shaping factor of sustainable consumption behavior. Social exchange theory supports finding a kind of mutual benefit in the interaction between people and society to change people’s view of society (Islam et al., 2018). Consumer engagement is based on the relationship between consumers and the industry and maintains a long-term consumption habit by allowing consumers to understand the concept of sustainable consumption (Čapienë et al., 2021). Thus, we hypothesis that:

*H14: Contextual factors positively influence consumer engagement.*

Consumer engagement is a partial mediator in how social network marketing shapes consumer purchase intention (Piligrimienë et al., 2020). Research on sustainable consumption in the retail industry shows that market conditions guide consumer engagement, thus shaping their shameful consumption behavior (Bãlan, 2020). Here, we developed:

*H15: Consumer engagement mediates the effect of contextual factors on SCBOEI.*

#### Contextual factors, identity, and sustainable consumption behavior in the Chinese online education industry

As a new self-concept, self-identity significantly promote the interpretation of sustainable consumption behavior and strengthen these behaviors. Contextual cues positively affect personal identity, which has been confirmed in the impact on Sustainable purchase and reduction behavior of Chinese and Polish consumers (Dermody et al., 2018). Thus,

*H16: Contextual factors positively affect identity.*

The goal of social identity theory is to explain behavior. In the context of sustainable consumption, identity tends to be a psychological factor with an intermediary effect. The impact of external conditions on sustainable consumption behavior may not be direct. The intermediary effect of identity in the middle has been proved in many fields (Rise et al., 2010). The government, market, and education have imperceptibly shaped consumers’ identities. In this particular field, the degree of consumer identity determines sustainable consumption behavior (Dermody et al., 2018).

*H17: Identity mediates the relationship between the contextual factors and SCBOEI.*

#### Demographics variable and sustainable consumption behavior in the Chinese online education industry

In terms of sustainable consumption, a multilevel approach allows researchers to explore whether the effect of individual-level demographic, psychological, or social variables (e.g., gender, income, education level) potentially differ as a function of cultural or other contextual factors (Milfont and Markowitz, 2016). Socio-demographics compromise age, education, and knowledge will influence green food purchases. Demographic determinants, social influences, and environmental values explain their potential weight in spurring SCB (Carfora et al., 2019). Hence:

*H18. Demographic variable positively affects the SCBOEI.*

The study examined six variables in total, with the independent variables being value and context factors, the intermediate variables being identity, sustainable consumption attitudes, and consumer engagement, and the dependent variable being SCBOEI. The study examined six variables in total, with the independent variables being value and context factors, the intermediate variables being identity, sustainable consumption attitudes, and consumer engagement, and the dependent variable being SCBOEI. Table 1 outlines their definitions.

**TABLE 1:** Definition of variables in this study.

Variable	Definition
Value	Consumers perceive the functional interests, emotional hedonism, and contributions to themselves and society in the consumption process of online education, so they are willing to change their consumption mode and behavior.
Sustainable consumption attitude (SCA)	Users have a positive attitude toward sustainable issues (quality education, international cooperation, decent economy development) when consuming online education and recognize the sustainable consumption behavior of industry platforms, courses, and resources.
Identity	Consumers’ perceived self-esteem and social respect in the consumption of the online education industry tend to change the psychology of consumer behavior for local development.
Contextual factor (CF)	The external force to promote online education for consumers to change their consumption behavior is a multi-dimensional concept. Government, market, education, COVID-19 pandemic, and media can become a part of its scope.
Consumer engagement (CE)	When consumers perceive the benefits of the online education industry to themselves and society to make their behavior changes, consumer engagement usually involves the level of physical, cognitive and emotional relationships between consumers and organizations, products, brands, etc.
SCBOEI	Consumption behavior in the online education industry is conducive to realizing various sustainable development goals related to society, economy, education, and the environment.

## Materials and methods

### Sample

Table 2 shows the demographic detail. There is a formula *n* = *N\1* + *N(e)^2^* to calculate the sample size for a simple random sampling design, with a 5% level of precision and a 95% confidence level for the equation, where n is the sample size; e specifies the desired level of precision, where e = 0.05; N is the population size. There are 1,014,764 students studying in tertiary institutions. Furthermore, they are in four types of tertiary institutions, including private junior colleges (14.8%), private undergraduate (16.1%), public junior colleges (27.3%), and public undergraduate (41.8%). According to this formula, 400 or so samples are calculated as the most suitable sample size for research. To ensure the reliability of the research, we have added some samples to different levels of institutions and finally adjusted the sample size to 440 at least.

**TABLE 2:** Demographic statistical analysis.

		Frequency	Percent
Gender	Male	265	47.4
	Female	294	52.6
Education level	Under the bachelor	207	8.6
	Bachelor	259	31.5
	Master	48	47.8
	Doctor	45	12.2
Family income	<$ 1,000	48	8.6
	$ 1,000–$ 2,000	176	31.5
	$ 2000–$ 4000	267	47.8
	>$ 4,000	68	12.2
Age	18–20	54	9.7
	21–25	126	22.5
	25–30	245	43.8
	>30	134	24.0

An Internet survey with 559 students in higher education institutions from Chongqing was conducted for 1 month using a convenient sampling method (Etikan et al., 2016). Convenient sampling is suitable for cases where the overall number is small and the individual differences are slight, reflecting a randomization principle. The subjects of the survey are all college students, with certain similarities. The overall number is 559, not too large a population. Thus, convenient sampling is more suitable for this study. Two hundred and sixty-five women and 294 men participated in the survey. Bachelor’s and vocational universities are the main participants, accounting for 83.3%, and the participants of master’s and doctoral students are relatively few, only 16.7%. Participants’ household income is mainly concentrated between $ 2,000 and $ 4,000, including 176 participants with a monthly household income of $ 1,000–$ 2,000, accounting for 31.5%, and 267 participants with $ 2,000–$ 4,000, accounting for 47.8%. Household monthly income of $ 1,000 is only 8.6%. Participants between the ages of 18–25 accounted for 95.5%, 15 participants between the ages of 25–30, only 10 over the age of 30, and 4.5% over the age of 25.

### Instrument

The survey questionnaire comprises seven parts. The items for SCBOEI, value, sustainable consumption attitude, identity, consumer engagement, and contextual factors are designed in the 1–6 parts. The last part is the data collection of the basic profiles of participants. The instruments were translated into Chinese (and back-translated into English for validity verification). To ensure the structural validity of the questionnaire, a confirmatory factor was analyzed, and multiple indicators were considered, such as the model fitting indices, chi-square to degrees of freedom (*X^2^/DF*), compare the fitting index (CFI), approximate root means square error (RMSEA), 95% confidence interval (90% CI) of RMSEA.

### Measurement of variables

Lin and Huang (2012) and Piligrimienë et al. (2020) have contributed to sustainable consumption behavior. This article rewrites the measurement of sustainable consumption behavior and value based on the value they discussed. Dermody et al. (2015) measured the identity and discussed the impact of social identity, self-identity, and place identity on sustainable consumption behavior. Therefore, the study adopted the scales adapted to this study by rewriting their choice of different items from the measures of identity. Zakaria et al. (2019) research and measurement of sustainability attitudes provide clues to the topic design for this article. The study measures consumer engagement by Piligrimienë et al. (2020). Contextual factors are based on Figueroa-García et al. (2018) measurement of government behavior, market conditions, and education. Table 3 shows the detail of the measurement source.

**TABLE 3:** Introduction to the source of the scale.

Variables	References
SCBOEI	Lin and Huang, 2012; Piligrimienë et al., 2020
Value	Lin and Huang, 2012; Biswas, 2017
Identity	Dermody et al., 2015
Sustainable consumption attitude (SCA)	Zakaria et al., 2019
Consumer engagement (CE)	Piligrimienë et al., 2020
Contextual factors (CF)	Figueroa-García et al., 2018

## Data analysis

After collecting the data, the questionnaire will be edited and coded. All questionnaires will be returned in numerical order. The data will then be analyzed using SPSS 22 version. In order to ensure that the data are accurately transcribed, the frequency of the data will be calculated before analyzing the data, so as to avoid data error or loss. Amos 26.0 can provide a graphical context for each step in the process of building equation models. *This study conducted descriptive analysis, confirmatory factor analysis, and path analysis*. Because each tool has different functions when processing multi-stage data, in this part, we will use these tools to process the data. First, the measurement model was tested, followed by confirmatory factor analysis (CFA) to evaluate the structural validity of the measurement. Next, SEM was tested to test hypothetical relationships proposed in the model. When evaluating model fitting, various fitting indicators (i.e., *x^2^/DF, CFI, GFI, AGFI, RMSEA*) were used. Finally, the bootstrapping method was conducted to test the mediating role of social identity as appeared in the model.

### Reliability and validity

SPSS 22.0 verified the overall reliability and validity of the study. Traditionally, if Cronbach’s α value is between 0.80 and 0.90, it indicates that it is perfect, and if the αvalue is 0.70 to 0.80, it indicates that it is pretty good. Between 0.65 and 0.70, the α value indicates the minimum acceptable. If α is in the range of 0.60∼0.65, there is no need to use it (Vaske et al., 2017). Therefore, the reliability survey of 31 questions shows that the value of Cronbach’s α is 0.946, indicating that the questionnaire has relatively good reliability, as shown in Table 4.

**TABLE 4:** Reliability statistics.

Cronbach’s alpha	Cronbach’s alpha based on standardized items	*N* of items
0.946	0.946	31

Through SPSS 22 analysis, *P-*value is less than 0.05, indicating that the questionnaire data are suitable for factor analysis. If the Kaiser-Meyer-Olkin measure of sampling adequacy (KMO) value is higher than 0.8, it indicates that the validity is high. If the KMO value is between 0.7 and 0.8, the validity is good. If the KMO value is between 0.6 and 0.7, the validity is acceptable. If the KMO value is less than 0.6, the validity is poor (Moore and Kirkland, 2007). Table 5 shows that *P* = 0.000 and KMO is 0.944, indicating that the questionnaire has good validity, which means that the questionnaire is quite suitable for factor analysis.

**TABLE 5:** Kaiser-Meyer-Olkin measure of sampling adequacy (KMO) and Bartlett’s test.

Kaiser-Meyer-Olkin measure of sampling adequacy.		0.944
Bartlett’s test of sphericity	Approx. Chi-square	9224.939
	Df	465
	Sig.	0.000

### Descriptive analysis

The average values of the 31 questions are 5.0–6.0, which altogether present a normal distribution, reflecting that the participants’ attitudes toward all dimensions are relatively moderate. They are not particularly able to accept and adapt in some aspects, nor are they particularly opposed or not adapted in some aspects. In other words, all dimensions may be helpful in the sustainable consumption behavior of consumers in the online education industry.

### Confirmation factor analysis

Amos is an essential tool for performing confirmatory factor analysis, which helps this study conduct confirmatory factor analysis on six measurement models. If the questionnaire title has a clear source and is demonstrated, Std ≥ 0.6 is usually required. If the questionnaire is rewritten or designed by ourselves, Std ≥ 0.5 indicates that the factor load can meet the verification factor analysis results (Moore and Kirkland, 2007). The chart shows that the Std of all items exceeds 0.6 and meets the requirements of validation factor analysis.

*X^2^/df* is smaller means the higher the fit between the hypothetical model and the observed data, and the larger the *X^2^/df*, the worse the model’s fit. Generally, the *X2/df* is less than 3, which means the model is well-adapted. RMSEA represents the square root of the approximation error of the comparison fitting index. Generally, *RMSEA* > *0.1* indicates that the fitting degree of the model is not good; 0.08–0.1 indicates that the model is general and has a common adaptation; 0.05–0.08 indicates that the model fits well. *RMSEA* < *0.05* indicates that the fitting degree of the model is excellent. For other fitting indexes, such as goodness of fit index (GFI), comparative fitting index (CFI), cumulative fitting index (IFI), and relative fitting index (TLI), the range of index data values is between 0 and 1. The closer the index is to 1, the better the fitting degree of the model is (Moore and Kirkland, 2007).

After testing, *X^2^/df* is *2.109 (<3)*, and the result is ideal. The values of GFI, AGFI, IFI, TLI, and CFI are more significant than or close to 0.90, respectively. The RMSEA index is 0.045, less than 0.08, indicating that there is an excellent model fit. As shown in Table 6.

**TABLE 6:** Confirmatory factor model fitting index.

Index	χ2/df	RMSEA	GFI	AGFI	IFI	TLI	CFI
Standard	<3	<0.08	>0.9	>0.9	>0.9	>0.9	>0.9
Results	2.109	0.045	0.910	0.894	0.948	0.942	0.948

According to Table 7, the standardized factor load of each item on the latent variable is more than 0.7 or close to 0.7, indicating that the observed variable has a reasonable explanation for the latent variable. The average variance of each variable is between 0.501 and 0.601, the AVE value reaches the required level of 0.5, and the combination reliability CR is between 0.817 and 0.883, all of which exceed 0.7, indicating that the aggregation validity is reliable (Hox and Bechger, 1998).

**TABLE 7:** Aggregation validity test.

Latent variable	Items	*N*-std	St error	C.R.	*P*	Std	CR	AVE
SCBOEI	Q1	1.000				0.757	0.876	0.542
	Q2	1.033	0.063	16.418	***	0.697		
	Q3	0.948	0.051	18.576	***	0.780		
	Q4	0.906	0.056	16.035	***	0.682		
	Q5	0.973	0.052	18.593	***	0.780		
	Q6	0.906	0.054	16.909	***	0.716		
Value	Q7	1.000				0.746	0.863	0.513
	Q8	1.047	0.059	17.840	***	0.775		
	Q9	1.055	0.062	17.044	***	0.742		
	Q10	1.171	0.071	16.445	***	0.717		
	Q11	0.996	0.070	14.335	***	0.629		
	Q12	1.087	0.070	15.504	***	0.678		
SCA	Q13	1.000				0.696	0.833	0.501
	Q14	1.023	0.064	15.869	***	0.772		
	Q15	0.782	0.058	13.429	***	0.638		
	Q16	0.984	0.067	14.771	***	0.709		
	Q17	0.959	0.065	14.859	***	0.714		
Identity	Q18	1.000				0.810	0.883	0.601
	Q19	0.989	0.051	19.422	***	0.757		
	Q20	1.015	0.048	21.048	***	0.806		
	Q21	0.966	0.049	19.534	***	0.761		
	Q22	1.028	0.055	18.845	***	0.740		
CE	Q23	1.000				0.729	0.875	0.583
	Q24	1.038	0.063	16.557	***	0.734		
	Q25	1.075	0.061	17.704	***	0.786		
	Q26	1.084	0.061	17.763	***	0.788		
	Q27	1.147	0.065	17.525	***	0.778		
CF	Q28	1.000				0.753	0.817	0.528
	Q29	0.842	0.058	14.421	***	0.654		
	Q30	0.980	0.059	16.659	***	0.759		
	Q31	0.962	0.059	16.179	***	0.735		

***p < 0.005.

In Table 8, the discriminant validity shows that the absolute value of the correlation coefficient of each latent variable is less than the square root of the average variance extraction amount AVE of the corresponding latent variable. That is, there is a certain degree of discrimination between the variables. Thus, the discriminant validity of the scale is reliable.

**TABLE 8:** Discriminant validity test.

Latent variable	1	2	3	4	5	6
SCBOEI	0.736					
Value	0.663	0.716				
SCA	0.654	0.606	0.708			
Identity	0.690	0.644	0.519	0.775		
CE	0.659	0.599	0.536	0.628	0.763	
CF	0.641	0.563	0.473	0.691	0.545	0.726

The diagonal is the square root of the corresponding dimension AVE.

### Path analysis

As mentioned above, the structural equation model analysis shows the value is χ^2^/df = 2.183 (<3), and the result is ideal. The values of GFI, AGFI, IFI, TLI, and CFI are more significant than or close to 0.90, respectively. The RMSEA index is 0.046 (<0.08), indicating that the model has good fitting, as shown in Table 9.

**TABLE 9:** Structural equation model fitting index.

Fitting index	χ2/df	RMSEA	GFI	AGFI	IFI	TLI	CFI
Standard	<3	<0.08	>0.9	>0.9	>0.9	>0.9	>0.9
Result	2.183	0.046	0.906	0.890	0.944	0.939	0.944

After establishing the structural equation model, Amos fitting calculation shows the non-standardized path coefficient, standard error S.E., standardized path coefficient, critical proportional value C.R., and significance *p*-value between variables after nine iterations. Generally, if the C.R. value is more significant than 1.96 and the *p*-value is less than 0.05, it can be considered that the path coefficient can pass the significance test within the 95% confidence interval, indicating that the corresponding path hypothesis of the present model is tenable; otherwise, the hypothesis is not tenable. Table 10 shows the results of the structural equation model path analysis.

**TABLE 10:** Structural equation path test.

Path	Estimate	Std-estimate	S.E.	C.R.	*P*	Hypothesis	Results
Value–>Identity	0.460	0.384	0.059	7.785	***	H2	Support
CF–>Identity	0.482	0.493	0.052	9.336	***	H16	Support
Value–>SCA	0.563	0.504	0.068	8.242	***	H5	Support
CF–>SCA	0.193	0.211	0.051	3.773	***	H12	Support
Value–>CE	0.476	0.438	0.061	7.837	***	H8	Support
CF–>CE	0.292	0.329	0.048	6.025	***	H14	Support
Value–>SCBOEI	0.159	0.141	0.070	2.286	0.022	H1	Support
CF–>SCBOEI	0.163	0.177	0.056	2.923	0.003	H11	Support
Identity–>SCBOEI	0.201	0.214	0.057	3.540	***	H3	Support
SCA–>SCBOEI	0.269	0.267	0.050	5.342	***	H7	Support
CE–>SCBOEI	0.216	0.208	0.052	4.191	***	H9	Support

***p < 0.005.

**FIGURE 1:**
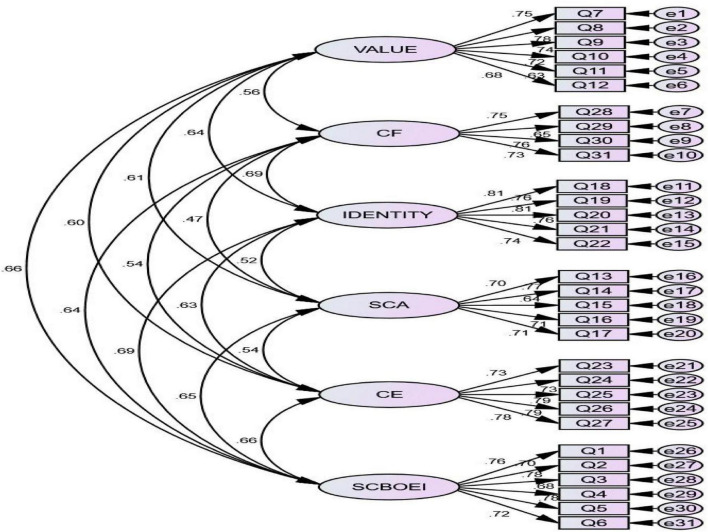
Confirmation factor analysis diagram.

The path analysis diagram is shown in Figure 1.

**FIGURE 2:**
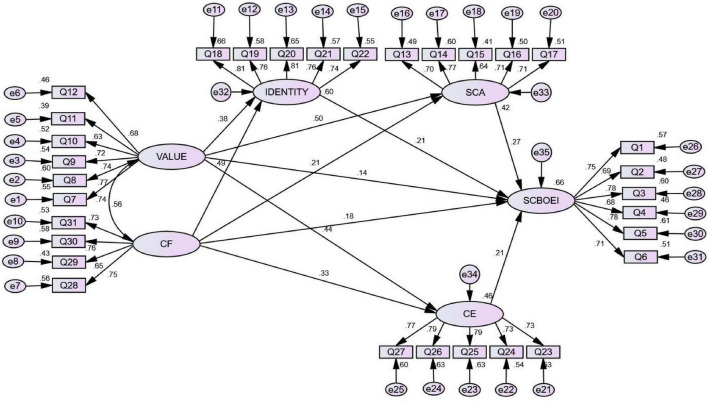
Path analysis for the model.

According to Figure 2, the path inspection results are as follows:

Value has a significantly positive effect on identity (Std = 0.384, *P* < 0.001).

CF has a significantly positive effect on identity (Std = 0.493, *P* < 0.001).

Value has a significantly positive effect on SCA (Std = 0.504, *P* < 0.001).

CF has a significantly positive effect on SCA (Std = 0.211, *P* < 0.001).

Value has a significantly positive effect on CE (Std = 0.438, *P* < 0.001).

CF has a significantly positive effect on CE (Std = 0.329, *P* < 0.001).

Value has a significantly positive effect on SCBOEI (Std = 0.141, *P* < 0.05).

CF has a significantly positive effect on SCBOEI (Std = 0.177, *P* < 0.05).

Identity has a significantly positive effect on SCBOEI (Std = 0.214, *P* < 0.001).

SCA has a significantly positive effect on SCBOEI (Std = 0.267, *P* < 0.001).

CE has a significantly positive effect on SCBOEI (Std = 0.208, *P* < 0.001).

The study put demographic variables into the model and tested them separately. In terms of demographic variables, only age influences SCBOEI, and others have no significant effect, as shown in Table 11.

**TABLE 11:** Path analysis between the demographics and sustainable consumption behavior in the Chinese online education industry (SCBOEI).

Path	Estimate	Std-estimate	S.E.	C.R.	*P*
Gender–>SCBOEI	–0.073	–0.049	0.046	–1.582	0.114
Education–>SCBOEI	–0.028	–0.029	0.038	–0.740	0.459
Income–>SCBOEI	0.005	0.005	0.045	0.107	0.915
Age–>SCBOEI	0.077	0.094	0.036	2.164	0.031

Table 11 inspires:

Gender has no significant effect on SCBOEI.

Education has no significant effect on SCBOEI.

Income has no significant effect on SCBOEI.

Age has a significantly positive effect on SCBOEI (Std = 0.094, *P* < 0.05).

Thus, H18 is partly supported.

### Indirect effect analysis

Applying the Bootstrap method of multi-mediation analysis, the structural equation software automatically outputs the estimated value of the mediation effect, corresponding standard error, *t*-value, and the significance of the mediation effect can be judged based on this information (Jung et al., 2019). The Bootstrap method requires no normality assumptions, large samples, and standard errors when estimating a mediation effect interval (indicating that the mediation effect is significant if the interval does not include 0) (Larriba et al., 2018). The bootstrapping method is used to test the mediating effect. The confidence interval is 95%. In this confidence interval, if the effect value does not exceed 0, it indicates that it has a significant mediating effect (Moore and Kirkland, 2007). Table 12 describes the detail of indirect effects results.

**TABLE 12:** Intermediary effect test.

Effect category	Path	Effect	Std-error	Bootstrapping		Percent	Hypothesis	
				95% CI				
Total effect	Value->SCBOEL	0.449	0.059	0.321	0.557			
Direct effect		0.141	0.072	0.008	0.291	31.40%		
Indirect effect	Value->Identity->SCBOEI	0.082	0.027	0.037	0.147	18.26%	H4	Support
	Value->SCA->SCBOEI	0.135	0.034	0.076	0.211	30.07%	H6	Support
	Value->CE->SCBOEI	0.091	0.026	0.046	0.152	20.27%	H10	Support
Total effect	CF->SCBOEI	0.408	0.06	0.291	0.522			
Direct effect		0.177	0.061	0.053	0.297	43.38%		
Indirect effect	CF->Identity->SCBOEI	0.105	0.035	0.044	0.179	25.74%	H17	Support
	CF->SCA->SCBOEI	0.056	0.019	0.025	0.102	13.73%	H13	Support
	CF->CE->SCBOEI	0.069	0.022	0.031	0.118	16.91%	H15	Support

(Source: by this study).

The 95% upper and lower intervals of the “value –> identity –> SCBOEI” path are [0.037, 0.147], excluding 0, indicating that identity plays a significant mediating role between value and SCBOEI, H4 is supported.

The 95% upper and lower interval of the “value –> SCA –> SCBOEI” path is [0.076, 0.211], excluding 0, indicating that SCA plays a significant intermediary role between value and SCBOEI, H6 is supported.

The 95% upper and lower interval of the “value –> CE –> SCBOEI” path is [0.046, 0.152], excluding 0, indicating that CE plays a significant intermediary role between value and SCBOEI, H10 is supported.

The 95% upper and lower intervals of the “CF –> identity –> SCBOEI” path are [0.044, 0.179], excluding 0, indicating that identity plays a significant mediating role between CF and SCBOEI, H17 is supported.

The 95% upper and lower intervals of the “CF –> SCA –> SCBOEI” path are [0.025, 0.102], excluding 0, indicating that SCA plays a significant mediating role between CF and SCBOEI, H13 is supported.

The 95% upper and lower intervals of the “CF –> CE –> SCBOEI” path are [0.031, 0.118], excluding 0, indicating that CE plays a significant intermediary role between CF and SCBOEI, H15 is supported.

## Results

Based on the factor analysis and path analysis for 559 valid questionnaires, the study proves that value, identity, attitude, engagement, and context factors are important pre-factors affecting SCBOEI. The overall identity, sustainable consumption attitude, and engagement play intermediary roles in the relationship between value and SCBOEI. The same situation also exists in the relationship between contextual factors and SCBOEI. However, the six mediating relationships explored in the study are partial. The mediating relationship of identity in value and SCBOEI is only 18.26%, and the mediating role of identity between contextual factors and SCBOEI accounts for 25.74%. The mediating effect of sustainable consumption attitude is 30.07% in the relationship between value and SCBOEI and only 13.73% between contextual factors and SCBOEI. The mediating effect of consumer engagement is 20.27 and 16.91%, respectively. In addition, the study also found that only age has a significant relationship with SCBOEI, while the relationship between education, family income, gender, and SCBOEI is not significant, which is different from the previous literature.

## Discussion and implications

The online education industry faces unprecedented development opportunities with a large number of consumers and market prospects (Xue and Li, 2021). The development of network education in Chongqing has been supported by policy, investment environment, and urban population in higher education, which has considerable advantages (Jiang and Pu, 2021).

The study of sustainable consumption behavior covers many fields (Wang and Yu, 2021). With the COVID-19 pandemic and the digital economy, the online education industry in Chongqing has developed rapidly and once became the main leasing force of major business centers (Chen et al., 2018; Jiang and Pu, 2021). With the support of policy, economy, and population welfare, the online education industry in Chongqing has excellent potential (Jiang and Pu, 2021). The study on SCBOEI in Chongqing has focused on many topics, such as paperless learning, education equality, education sharing, gender equality, employment, digital technology, and learning environment (Jiang and Pu, 2022). This study provided further suggestions for various stakeholders.

### Theoretical implications

The theory of sustainable consumption behavior has long paid attention to the transformation of consumption patterns in the fields of energy, nature, and economy, especially to explore consumers’ contribution to the goal of sustainable development in these traditional fields (Wang and Yu, 2021). The research provides new thinking for sustainable consumption behavior and breaks through the traditional education for sustainable development theory. Online education can be one of the crucial products of the digital era and the era of sharing economy (Mishra et al., 2020). The era changes brought by knowledge sharing are not satisfied by traditional education. Many online education discussions involve technology development, curriculum construction, and teacher allocation (Jiang and Pu, 2022). As a basic form and prosperous development trend in the digital era, online education should be its strength that can serve sustainable development (Dong et al., 2021). Therefore, exploring SCBOEI provides a new paradigm for education for sustainable development, makes up for the gap in the sustainable development of online education services, and has prominent theoretical significance.

Sustainable consumption behavior includes three dimensions: society, economy, and environment (McEachern et al., 2020). The most studied field of sustainable consumption behavior is the discussion of the environment, such as green economy, green food, and resource protection (Wang and Yu, 2021).

The study constructed 18 hypotheses and proved that these hypotheses are tenable. Moreover, the research is placed in the network education industry, which has developed relevant theories. The research integrates functional value, social value, epistemic value, emotional value, conditional value, and epistemic value to form the research dimension of value. It is found that value can predict SCBOEI.

The attitude-behavior-context theory explains the SCB in the energy industry and makes the model highlight the role of attitude and context in the SCB (Xu et al., 2017). The research breaks through the previous impact of environmental attitude on sustainable consumption behavior and determines the role of sustainable consumption attitude in SCBOEI. Research on sustainable consumption attitudes has been carried out in many fields, such as tourism (Kosonen and Kim, 2016). The sustainable consumption attitude of online education provides suggestions and references for the sustainable consumption behavior of other industries.

Social exchange theory attempts to solve the problem of mutual benefit in human and sustainable development and has achieved specific results in both enterprises and individuals (Wang et al., 2019). The research further proves the impact of engagement on sustainable consumption behavior and determines the intermediary role of engagement in the relationship between contextual factors and SCBOEI. The research integrates several theoretical premises, and the SCBOEI model introduces the influence of internal and contextual factors on SCBOEI.

Undoubtedly, the research has developed and improved the sustainable consumption theory, but the relevant research results revealed some inspiration and suggestions for the market, consumers, government, educational institutions, and other stakeholders.

### Practical implications

Almost the world is encouraging measures to promote sustainable consumption. The results of the education for sustainable consumption study give many suggestions to schools, markets, and government departments (Ruby et al., 2020).

Sustainable consumption behavior in the Chinese online education industry pays attention to the sustainable development of the economy and education. Knowledge sharing, quality education, lifelong education, gender equality, and international cooperation are the goals that education in the county has the opportunity and sufficient ability to achieve and complete. Therefore, all sectors of society, especially education and government departments, should pay attention to the impact of the online education industry on student consumers and actively use these trends to popularize the knowledge of SCBOEI.

The network education market’s policy, technology, investment, and consumption are becoming increasingly intensive (Xue and Li, 2021). These results provide market personnel with opportunities. They also suggest that marketers actively seize the development opportunities of the online education industry and adopt the operation mode of leading technology and leading service (Gómez-Zermeño, 2020). The government also issued a large number of policies of supervision, regulation, and support. Schools are also actively responding to the development trend of online education, encouraging students and teachers to adapt to digital education’s development (Dong et al., 2021).

Higher education students have found the advantages of online education. In addition, in the discussion of value, the research reveals that consumers realize that in the era of sharing, their consumption behavior has paved the way for sharing gender equality and quality education for sustainable development. However, through the efforts made for social development, especially international cooperation and exchange of education in remote areas, consumers will be more willing to choose online education.

The government and higher education institutions are responsible for popularizing technology’s significance to sustainable development in the digital era (Figueroa-García et al., 2018). It inspires us should include the introduction of relevant content on sustainable consumption behavior in online education, so that the whole student consumer group can popularize the consumption concept of sustainable development, actively change the consumption model, and serve sustainable development. Schools and families should shape the student’s identity for sustainable development, which is the leading force in shaping knowledge in this area (Adam et al., 2021).

The online education market should actively use various methods to cultivate identity. Moreover, the marketing department should use the clues provided by the research to formulate marketing policies conducive to cultivating identities to promote industry development. At the same time, government and the market should actively create online education for local brands to serve the development of the local economy, which can make consumers feel that the consumption of online education has a positive impact on the development of local economy and education.

The research can also encourage consumers to create online education communities to encourage everyone to share relevant information to achieve identity, encourage people to have more consistency, and get mutual support in using online education to achieve SDGs. Government departments are also responsible for encouraging the development of online education communities, providing open social teachers, serving the development of community education, promoting consumer recognition, helping them form more sustainable development ideas, and creating a sustainable consumption atmosphere.

Consumers should actively cultivate a sustainable consumption attitude in the online education industry, education department, or business field. Similarly, higher education institutions have sufficient responsibility and reason to explain the impact of a sustainable consumption attitude on sustainable consumption behavior. We should increase investment in talents, platforms, and resources regarding sustainable consumption education. Keep up with the pace of digital and intelligent education, and actively use digital technology to promote the sustainable development of online education services.

The consumer engagement refers to obtain consumer recognition and insistence in this field. For example, to improve product quality, improve after-sales, and increase product creativity (Piligrimienë et al., 2020). The study reveals consumers’ possible obsession with online education brands and platforms from this variable. However, this inherent thinking is difficult to change for consumers. Therefore, the market should have positive brand guidance and use relevant, sustainable development reasons to support consumers in making decisions and realizing the transformation of consumer behavior into subtle consumption.

Context factors predict sustainable consumption behavior and suggest that social media, government, and schools become significant contributors (Figueroa-García et al., 2018). The study discusses the positive impact of contextual factors on SCBOEI from the perspectives of government, market, and education. The research shows that the government can realize that policies or actions play a significant role in SCBOEI, so the government should guide sustainable consumption when paying attention to the online education industry. At the same time, the policies and publicity of the online education market should be closely combined with the concept of sustainable development to help the online education industry move forward steadily and provide its consumers with more diverse and sustainable consumption patterns. Sustainable education is a factor that has been demonstrated in various contexts and positively impacts SCBOEI. Therefore, schools play an essential role. Teachers, students, and courses should pay attention to sustainable development, especially the sustainable consumption behavior of online education in curriculum design and practical activities.

Age, gender, income, and education always impact sustainable consumption in all areas (Soltani et al., 2020). However, this study only reveals the effect of age on SCBOEI, and the research can provide the corresponding reference for school education. The research can provide the corresponding reference for school education. Finally, institutions and markets need to pay attention to consumption behavior at different ages, and the research shows that age is positively correlated with SCBOEI. Therefore, higher education institutions should pay special attention to lower-grade students.

To sum up, understanding SCBOEI and the relevant factors can become the basis for government policy-making. At the same time, the research conclusion is also a reference for market development and standardization. Market researchers can highlight sharing and public welfare in product development and design, platform promotion, and after-sales links to attract consumers to change their consumption mode and form a sustainable consumption atmosphere. Undoubtedly, the research findings can be applied to the sustainable education of higher education, make more students realize the sustainable development of new fields, and make students in the digital age understand the importance of SCBOEI. As the mainstream consumer of online education, the research will bring them much thinking.

## Limitation of the study and suggestion for future research

### Limitation of the study

Our study has a number of limitations that open up potential research avenues. When studying the sustainable consumption behavior of online education, this article studies and analyzes the psychological and contextual factors. However, it puts forward the value, identity, attitude, engagement, and contextual factors on the comprehensive basis of multiple theories, constructs a theoretical research model, and makes an empirical analysis. The explanations of value, sustainable consumption attitude, and contextual factors are only considered for the research. There are many influencing factors of sustainable consumer behavior, and the research does not include them. Therefore, this article has some limitations in the research. Religion, culture, world views, and other factors related to sustainable behavior research are not considered. Nevertheless, future research can further extend SCBOEI research by employing a finer-grained analysis in investigating the antecedents and outcomes.

In this article, when studying SCBOEI, there is no in-depth study on population variables. The main reason is that researchers believe that higher education students are a changeable group. The buyers and users of online education are misplaced in their roles. At present, most of the respondents are undergraduates. Most online education users are not only higher education groups but also good at sharing and using. Therefore, this article has some limitations in the research. There is ample room in investigating different segments and customer groups.

The data samples collected in this article also have some limitations. Although researchers try their best to collect data, the main population is undergraduates, which cannot cover too many groups above and below undergraduates. Therefore, the research conclusion is one-sided, which only reflects the SCBOEI in the higher education stage or is not common in the stage of K12 education. Finally, online education is a product closely related to technology, which has an intense color of digitization and knowledge sharing. Therefore, the research does not consider the content of technical variables, which may need to be adjusted further in some aspects.

### Implications for future research

First, the current research mainly demonstrates the role of psychological and contextual factors on SCBOEI. However, each variable is comprehensively demonstrated in quantitative research. Therefore, future research can not only pay attention to the impact of religion, belief, culture, and race on SCBOEI but also concretize each variable and explore the impact of sub-variables on SCBOEI to construct a grand SCBOEI system, which lays a foundation for building a more detailed SCBOEI in future.

Second, the study of SCBOEI can be extended to the whole world, even in cross-regional and cross-age groups. For example, comparing SCBOEI between urban and rural consumer groups and similar comparisons can also be made in different countries and cities. SCBOEI research with different cultural backgrounds is conducive to improving the world significance and macro nature of SCBOEI research. Moreover, the research can focus on the K12 online education market and explore consumer behavior from product and service providers. Furthermore, this article only investigates the samples in Chongqing. While our theoretical arguments are generalizable to other mainland liberalized cities or regions in China, these are rather interesting findings in that our results may not apply to the entire country of China, such as Hong Kong or Macao, which share distinct regional culture. Future research is also encouraged to examine the SCBOEI in the cross-border and global context, because decision-makers share different emotions when engaging with international business (Dong, 2022).

Finally, the research can be discussed from the aspect of technology. There are not many research results of the *Technology Acceptance Model* in sustainable consumption behavior, but online education is an industry closely related to technology. In the era of sharing economy, online education for sustainable development is a new paradigm of educational development, and the educational innovation brought by technology deserves our attention. Therefore, discussing the technology acceptance model or its development model on SCBOEI should attract our attention to provide relevant suggestions for technology channels and promote the sustainable development of online education.

## Conclusion

In this study, we investigate factors affecting sustainable consumption behavior in the Chinese online education industry. This study first describes the prosperity and development of the online education industry in the context of the COVID-19 pandemic and digitization. Education for sustainable development is an essential focus in the twenty first century, and there is less discussion in the online education sector. Therefore, the research found this gap and decided to quantitatively study the sustainable consumption behavior of the online education industry.

Through confirmatory factor analysis, path analysis, and intermediary effect analysis, value and context factors are independent variables affecting SCBOEI, and identity, sustainable consumption attitude, and consumer engagement are intermediary variables in the model. Moreover, the study determined that among the demographic factors, only age has a significant impact on SCBOEI, and the explanatory effect of other demographic factors is relatively weak. Finally, the study confirmed the factors affecting SCBOEI and established the model diagram of SCBOEI. The research breaks through the explanation of a single theory on sustainable consumption behavior, combines consumer value theory, planned behavior theory, attitude-behavior-context theory, social identity theory, and social exchange theory for the first time, develops these theories in the online education industry, determines their explanatory role on SCBOEI, introduces the salient factors affecting the SCBOEI from different dimensions, and enriches the theory of sustainable consumption behavior.

This article puts forward some suggestions for the sustainable development of online education services. In the theoretical contribution of the research, the research puts forward suggestions to stakeholders. The research can help marketing practitioners in the online education industry formulate methods conducive to sustainable development, including strengthening publicity and constructing ideas. More importantly, these efforts will provide greater insights for government policymakers. They are also encouraged to construct sustainable development of online education services actively, actively integrate the concept of sustainable development into policies and investment, and guide consumers, significantly higher education students, to consume sustainably. The research has also inspired schools. In curriculum design, schools should emphasize sustainable development under the digital background, including how online education promotes the sustainable development of society, economy, and education and its contribution to human society. Students should also pay attention to the innovative role of online education in education or the education industry in the twenty first century. Finally, consumers also need to reflect on the consumption process, actively integrate into the sustainable consumption atmosphere, change the consumption model of the online education industry, and pay more attention to sustainable consumption behavior in the education industry to contribute to sustainable development. There are some limitations, including factors of incompleteness and population singularity, which make the research face problems of insufficient universality. Moreover, the research did not pay attention to the impact of technical factors on SCBOEI, which means that there are still many research gaps in the sustainable consumption behavior of online education under the digital background, which also leaves more space for future research.

## Data availability statement

The raw data supporting the conclusions of this article will be made available by the authors, without undue reservation.

## Author contributions

RP and SJ: conceptualization and research design. RP, SJ, and RD: methodology and formal analysis. RP, SJ, RD, and TC: visualization, supervision, and writing—original draft preparation. RP, SJ, RD, TC, AS, SR, and DT: writing—revising, and editing. All authors have read and agreed to the published version.
